# Conditionally replicating adenovirus prevents pluripotent stem cell–derived teratoma by specifically eliminating undifferentiated cells

**DOI:** 10.1038/mtm.2015.26

**Published:** 2015-08-12

**Authors:** Kaoru Mitsui, Kanako Ide, Akiko Takayama, Tadahisa Wada, Rie Irie, Ken-ichiro Kosai

**Affiliations:** 1 Department of Gene Therapy and Regenerative Medicine, Kagoshima University Graduate School of Medical and Dental Sciences, Kagoshima, Japan; 2 Center for Innovative Therapy Research and Application, Kagoshima University Graduate School of Medical and Dental Sciences, Kagoshima, Japan

## Abstract

Incomplete abolition of tumorigenicity creates potential safety concerns in clinical trials of regenerative medicine based on human pluripotent stem cells (hPSCs). Here, we demonstrate that conditionally replicating adenoviruses that specifically target cancers using multiple factors (m-CRAs), originally developed as anticancer drugs, may also be useful as novel antitumorigenic agents in hPSC-based therapy. The *survivin* promoter was more active in undifferentiated hPSCs than the *telomerase reverse transcriptase (TERT)* promoter, whereas both promoters were minimally active in differentiated normal cells. Accordingly, *survivin*-responsive m-CRA (Surv.m-CRA) killed undifferentiated hPSCs more efficiently than *TERT*-responsive m-CRAs (Tert.m-CRA); both m-CRAs exhibited efficient viral replication and cytotoxicity in undifferentiated hPSCs, but not in cocultured differentiated normal cells. Pre-infection of hPSCs with Surv.m-CRA or Tert.m-CRA abolished *in vivo* teratoma formation in a dose-dependent manner following hPSC implantation into mice. Thus, m-CRAs, and in particular Surv.m-CRAs, represent novel antitumorigenic agents that could facilitate safe clinical applications of hPSC-based regenerative medicine.

## Introduction

Human pluripotent stem cells (hPSCs), including human embryonic stem cells (hESCs) and human-induced pluripotent stem cells (hiPSCs), are promising sources of material for use in cell transplantation therapy. However, the risk of formation of tumors, including teratomas and cancers originating from contaminating undifferentiated and transformed cells, represents the most critical obstacle to the safe clinical application of hPSC-based regenerative medicine.^1^ Multiple approaches have been taken to improve safety by reducing the risk of carcinogenesis. However, most previous studies focused on improving generation of hiPSCs by eliminating potential oncogenic factors, such as the oncogene c-myc, or by integrating the reprogramming transgenes into chromosomes.^1^ Although these sorts of strategies, classified here as the first safety approach, reduce the reprogramming-associated oncogenic potential of hiPSCs, they cannot completely eliminate tumorigenic potentials due to the intrinsic characteristics of hPSCs, *i.e.*, self-renewal and pluripotency; consequently, it is still possible for teratomas to arise from contaminating undifferentiated hPSCs. In addition, chromosome instability, activation of oncogenic networks, and considerable plasticity are naturally prevalent in hPSCs, which accumulate genomic abnormalities during cell culture, possibly resulting in malignant transformation in some heterogeneous cells.^1^ Moreover, a recent study demonstrated that transient expression of reprogramming factors leads to cancer development in the absence of genomic abnormalities.^2^ In this regard, it is important to note the historical lessons of clinical gene therapy: in a number of preclinical animal studies, no tumorigenicity was observed, but there was nonetheless a high incidence of leukemia following *ex vivo* gene and cell-transplantation therapy in actual clinical trials.^3^ Thus, current preclinical studies, which allow experimental comparison of *in vivo* tumorigenic activities among different treatment groups, have insufficient sensitivity to guarantee clinical safety. In other words, in the context of first-in-human trials of innovative cell therapies, we cannot confidently predict that the risk of tumorigenicity has been eliminated. Consequently, innovative safety approaches should be developed in order to decrease this risk.

We previously developed a novel method (adenoviral conditional targeting) that securely isolated target cells from other cell types and undifferentiated hPSCs.^4^ This method, which can increase the efficacy and safety of hPSC-based regenerative medicine by decreasing tumorigenicity, is classified here as the second safety approach. Strategies that can directly target and kill, rather than merely inhibit, tumorigenic cells are classified here as the third safety approach. In this regard, a few recent studies have described generation of engineered hPSCs, in which a suicide gene under the transcriptional control of a pluripotency-related promoter is stably transduced.^5^ Although this approach reduces teratoma formation, the prevalent inactivation of an integrated transgene in undifferentiated hPSCs and the risk of carcinogenesis due to integration-derived mutagenesis suggest that this strategy may not be universally effective.^3,4^ Thus, there is an urgent need for a novel strategy that specifically kills tumorigenic undifferentiated hPSCs using a different methodology; we classify such future strategies as the fourth safety approach.

Conditionally replicating adenoviruses (CRAs), also called oncolytic adenoviruses, can selectively replicate in and kill cancer cells; consequently, CRAs represent attractive anticancer drugs.^6^ Previously, we developed a method for generating CRAs that can target cancers with multiple cancer-specific factors (m-CRAs); this approach further increased cancer specificity without reducing the anticancer effects.^7,8^ We also demonstrated that among candidate m-CRAs, *survivin*-responsive m-CRA (Surv.m-CRA) is one of the most promising anticancer agents, in two respects: superior cancer specificity (*i.e*., safety) and therapeutic efficacy relative to clinically tested telomerase-responsive m-CRAs (Tert.m-CRAs), and strong anticancer effects against currently incurable cancer stem cells (CSCs).^8–10^

Here, we show that the *survivin* promoter, like the *telomerase reverse transcriptase (TERT)* promoter, is highly activated in undifferentiated hPSCs, but is almost inactive in differentiated hPSCs and normal cells. Finally, we demonstrate that m-CRAs, in particular Surv.m-CRAs, are potentially useful as both potent anticancer drugs and as novel antitumorigenic agents in hPSC-based regenerative medicine; in the latter context, the viruses act by specifically killing contaminating undifferentiated hPSCs.

## Results

### High mRNA levels and promoter activities of survivin and TERT in undifferentiated hPSCs

**Figure 1:**
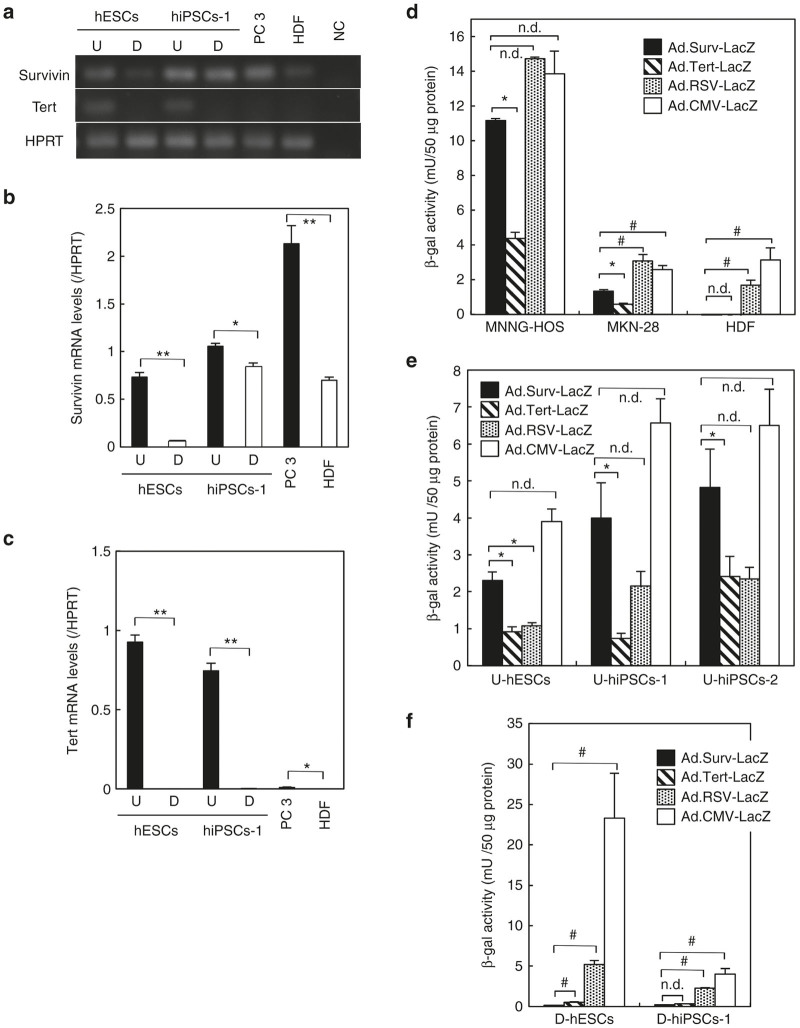
Endogenous mRNA expression and promoter activities of *survivin* and *TERT* in human pluripotent stem cells (hPSCs). (**a–c**) *Survivin* and *TERT* mRNA expressions in undifferentiated (U) and differentiated (D) states of hESCs and hiPSCs-1 (201B7), cancer cells (PC3), and differentiated normal cells (human dermal fibroblasts (HDFs)) were examined by reverse transcription–polymerase chain reaction (RT-PCR) (**a**) and accurately quantitated by qRT-PCR (**b,c**). The *HPRT* gene was amplified as an internal control. *n* = 4, each group. *P < 0.05 and **P < 0.01. (**d–f**) β-gal activity was measured 48 hours after infection with Ad.Surv-LacZ, Ad.Tert-LacZ, Ad.RSV-LacZ, or Ad.CMV-LacZ at a multiplicity of infection of 30 in cancerous (MNNG-HOS and MKN-28) or differentiated normal HDFs (**d**), undifferentiated hPSCs (hESCs, hiPSCs-1 (201B7), and hiPSCs-2 (253G1)) (**e**), and differentiated hPSCs (**f**). *n* = 3, each group. *P < 0.05 (higher in Ad.Surv-LacZ); #*P* < 0.05 (lower in Ad.Surv-LacZ); n.d., no statistical difference. hiPSCs, human-induced pluripotent stem cells.

Telomerase activity, expression levels, and promoter activities of *TERT* are high in both cancerous cells and in undifferentiated normal cells.^11,12^ On the other hand, expression levels and promoter activities of *survivin* are also high in cancerous cells,^8–10,13,14^ and a recent study suggested that *survivin* contributes to teratoma formation by hESCs.^15^ However, *survivin* promoter activity in undifferentiated normal cells has not yet been carefully examined. Using reverse transcription–polymerase chain reaction (RT-PCR) and quantitative real-time PCR (qRT-PCR) analysis, we found that endogenous *survivin* and *TERT* mRNAs were expressed at high levels in undifferentiated hESCs and iPSCs relative to differentiated cells, as well as in PC3 cancer cells relative to normal human dermal fibroblasts (HDFs); however, a low level of *survivin* mRNA expression was detected in normal HDFs and differentiated hiPSCs (Figure 1a–c).

The promoter assay demonstrated that both *survivin* and *TERT* promoters exhibited strictly cancer-specific activities (*i.e*., strong activity in cancer cells and undetectable activity in normal cells), and that the *survivin* promoter is stronger than the *TERT* promoter in cancer cells (Figure 1d), consistent with our previous studies on cancer.^9,16^ Moreover, the activity of the *survivin* promoter was very high, relative to the *TERT* promoter and Rous sarcoma virus long terminal repeat (RSV promoter), a representative ubiquitously strong promoter,^17,18^ in undifferentiated hPSCs but not in differentiated hPSCs (Figure 1e–f). Thus, the *survivin* promoter region that we use is able to strongly induce not only cancer-specific, but also undifferentiated cell-specific transactivation.

### Both Surv.m-CRA and Tert.m-CRA exhibit undifferentiated cell-specific replication and cytotoxicity in hPSCs

**Figure 2:**
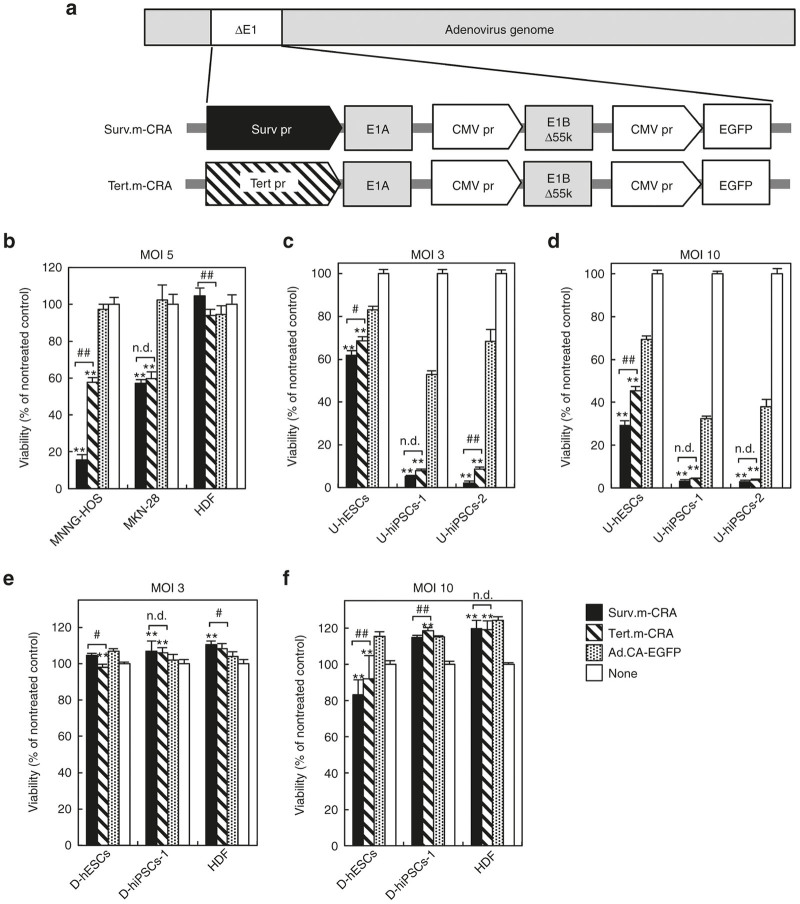
Cytotoxicity of m-CRAs in undifferentiated or differentiated human pluripotent stem cells (hPSCs) *in vitro*. (**a**) Schematic representation of the construction of both m-CRAs. (**b–d**) Cell viability was determined by WST-8 assay 4 days after infection with each virus at a multiplicity of infection (MOI) of 5 or 0 in cancerous (MNNG-HOS and MKN-28) and normal (human dermal fibroblasts) cells (**b**) or at an MOI of 3 or 10 in single undifferentiated hPSCs (**c** and **d**). (**e** and **f**) Differentiation was induced by generation of embryoid bodies, followed by cultures in suspension for 7 days, and in attached cultures for an additional 23 days. Differentiated hPSCs were infected with each virus 1 day after plating of single isolated cells. Consequently, the infected cells were cultured for 7 days, and cell viability was determined by the WST-8 assay. *n* = 8, each group. *P < 0.05 and **P < 0.005 (Surv.m-CRA or Tert.m-CRA versus the control, Ad.CA-EGFP); #*P* < 0.05 and ##*P* < 0.005 (Surv.m-CRA versus Tert.m-CRA); n.d., no statistical difference between Surv.m-CRA and Tert.m-CRA. CMV pr, CMV promoter; EGFP, enhanced green fluorescent protein; HDF, human dermal fibroblast; Surv pr, Survivin promoter; Tert pr, Tert promoter.

**Figure 3:**
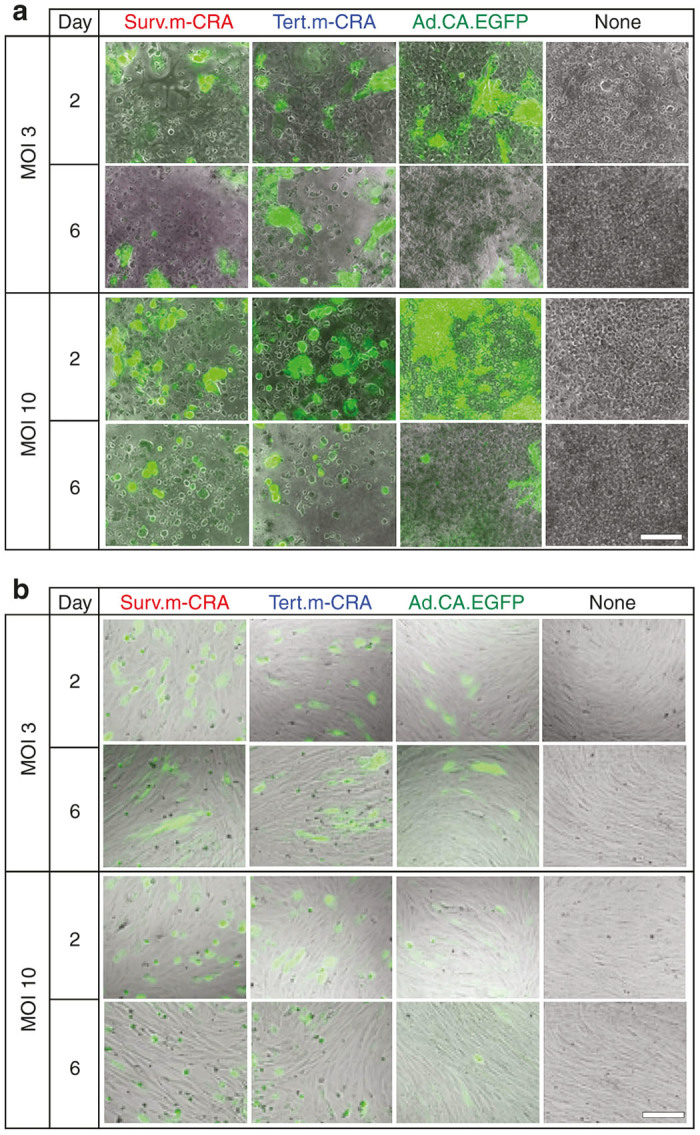
Microscopic images of undifferentiated or differentiated human embryonic stem cells (hESCs) after infection with each virus *in vitro*. Microscopic images were acquired by phase-contrast and fluorescence microscopy 2 and 6 days after infection of undifferentiated (**a**) or differentiated (**b**) hESCs. Each virus was infected at a multiplicity of infection of 3, 10, or 0. A number of dead cells were floating, and the remaining enhanced green fluorescent protein (EGFP) cells exhibited adenoviral cytopathic effects 6 days after infection of undifferentiated hESCs with Surv.m-CRA or Tert.m-CRA. By contrast, there was no apparent increase in either EGFP-expressing or dead cells after infection of differentiated hESCs with m-CRAs. Scale bar, 100 µm. MOI, multiplicity of infection.

In Surv.m-CRA and Tert.m-CRA, the adenoviral early region 1A (E1A) was regulated by promoters of *survivin* and *TERT*, respectively, and both viruses ubiquitously express enhanced green fluorescent protein (EGFP). We next investigated whether Surv.m-CRA and Tert.m-CRA exerted efficient and undifferentiated cell-specific viral replication and cytotoxicity in hPSCs, relative to two control groups infected with replication-deficient adenoviral vector ubiquitously expressing EGFP (Ad.CA-EGFP) or no transgenic protein (Figures 2 and 3). By microscopically observing the spread of virus-infected EFGP-expressing cells and the swollen dying cells that are a characteristic feature of the adenoviral cytotoxicity, both Surv.m-CRA and Tert.m-CRA induced prominent viral replication, resulting in cytotoxicity, in the undifferentiated states of all three hPSCs, as well as in two types of cancer cells, HOS-MNNG and MKN-28; these effects were dose-dependent (Figures 2b–d and 3). By contrast, Surv.m-CRA and Tert.m-CRA exerted no apparent viral replication, and undetectable or minimal cytotoxicity, in the differentiated states of hPSCs and in normal HDFs (Figures 2e,f and 3). Moreover, a decrease in the ratio of EGFP-expressing cells was observed 6 days after Ad.CA-EGFP infection only in undifferentiated hESCs, but not in the differentiated state, despite the absence of any cytotoxicity detectable under the microscope, suggesting that the episomal adenoviral transgene was diluted by cell division (Figure 3).

### Surv.m-CRA kills undifferentiated hPSCs more potently and specifically than Tert.m-CRA

**Figure 4:**
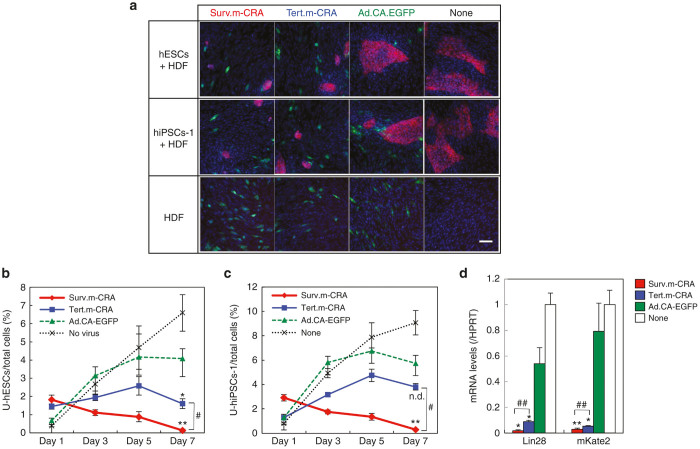
Viability of mKate2-expressing human pluripotent stem cells (hPSCs) cocultured with normal human dermal fibroblasts (HDFs) after infection with each virus *in vitro*. (**a**) hPSCs were cocultured with HDFs that had been plated 1 day before. One day after the hPSCs were plated, all the cells were infected together with each virus at a multiplicity of infection of 3 on day 0, followed by coculture under undifferentiated conditions for 7 days. As a control, HDFs alone (without hPSCs) were infected with each virus in the same manner on day 0. On day 7, representative fluorescence images, taken immediately after nuclear staining with Hoechst 33342, revealed virus-infected enhanced green fluorescent protein (EGFP)-expressing cells (green), mKate2-expressing hPSCs (magenta), and the nuclei of both types of cells (blue). Scale bar, 100 µm. (**b** and **c**) Nuclei (*i.e*., all cells) and mKate2-expressing human embryonic stem cells (hESCs) (**b**) or hiPSCs-1 (**c**) were counted using a CellInsight platform, and the percentages of hPSCs were calculated 1, 3, 5, and 7 days after infection. *n* = 4, each group. (**d**) mRNA expression levels of *Lin28* and *mKate2* on day 7 were examined by qRT-PCR. *n* = 8, each group. (**b–d**) Statistical analyses of the data on day 7. *P < 0.05 and **P < 0.005 (Surv.m-CRA or Tert.m-CRA versus Ad.CA-EGFP); #*P* < 0.05 and ##*P* < 0.005 (Surv.m-CRA versus Tert.m-CRA); and n.d., no statistical difference. hiPSCs, human-induced pluripotent stem cells; qRT-PCR, quantitative real-time PCR.

To accurately assess how each m-CRA specifically killed undifferentiated hiPSCs, but not differentiated normal cells, we cultured engineered hPSCs that stably expressed the far-red fluorescent protein *mKate2* on HDF cells, and analyzed the cell types that exhibited efficient viral replication and cytotoxicity after m-CRA infection (Figure 4). Infection with control Ad.CA-EGFP demonstrated that type 5 adenovirus could efficiently infect both hPSCs and HDFs (Figure 4a). Infection with each m-CRA significantly decreased only the number of far-red hPSCs, but not the number of HDFs, as time went on. The percentage of all cells (*i.e.*, blue-stained nuclei) on each dish that were far-red hPSCs was accurately determined by cell image analysis 1, 3, 5, and 7 days after infection. The results showed that hPSCs were more efficiently killed by Surv.m-CRA than by Tert.m-CRA (Figure 4b,c); this tendency was consistent with the results of the viability and promoter assays described above (Figures 2 and 3).

qRT-PCR analyses of *Lin28*, a representative pluripotency-associated gene,^19^ and *mKate2*, which was transduced and stably expressed in hPSCs but not HDFs, further supported the conclusion that the undifferentiated hPSCs were potently killed by both m-CRAs, and that Surv.m-CRA was a more potent killer than Tert.m-CRA (Figure 4d).

### Teratoma formation after hPSC implantation was inhibited by m-CRA pretreatment

**Figure 5:**
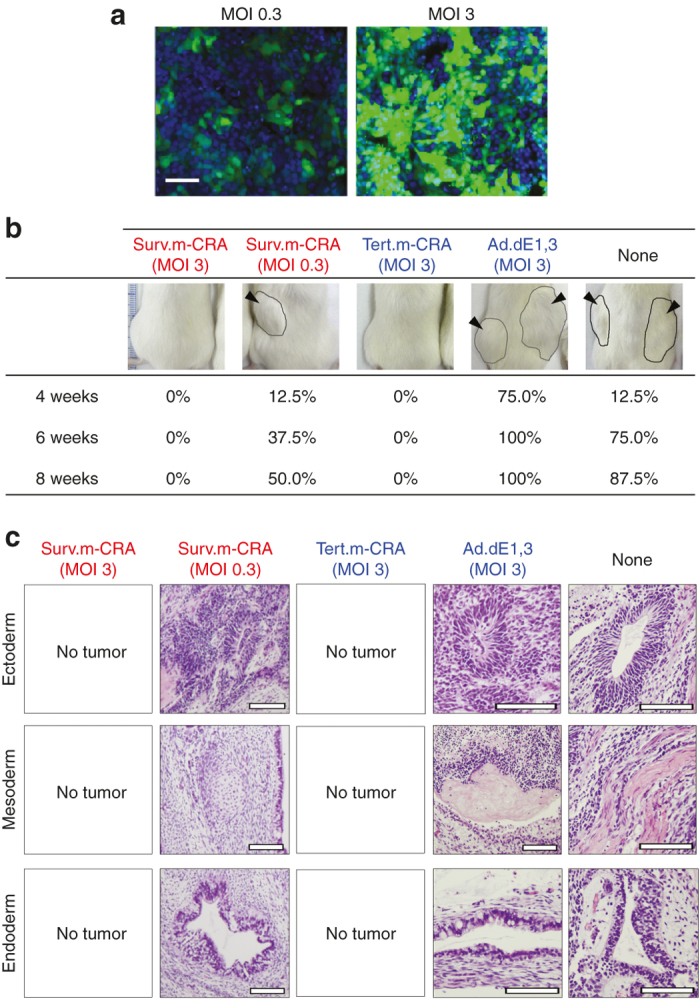
Antitumorigenic effects *in vivo*. (**a**) Fluorescence microscopic images were taken 48 hours after infection with replication-deficient Ad.CA-EGFP at a multiplicity of infection (MOI) of 0.3 or 3. The nuclei (blue) and enhanced green fluorescent protein (EGFP)-expressing human embryonic stem cells (hESCs) (green) were counted using the CellInsight platform, and the percentages of EGFP-positive hESCs were calculated. *n* = 8, each group. Scale bar, 100 μm (**b**) hESCs were infected with Surv.m-CRA at an MOI of 0.3 or 3, or Tert.m-CRA or Ad.dE1.3 at an MOI of 3 or 0, for 1 hour, and then 3.6 × 10^7^ of the infected cells were subcutaneously implanted into the dorsal flanks in severe combined immunodeficient mice. The percentages of mice with visible tumor nodules in each group 4, 6, and 8 weeks after adenovirus-infected hPSCs implantation, and representative macroscopic pictures 8 weeks after implantation, are shown (*n* = 8 mice per group). Lines and arrowheads indicate macroscopic tumor nodules. (**c**) Histopathological analysis of tumor nodules was performed 8 weeks after implantation of adenovirus-infected human pluripotent stem cells. Scale bar, 100 µm. hPSCs, human pluripotent stem cells.

Finally, we examined the efficiency with which the m-CRAs inhibited *in vivo* tumor formation after inoculation of undifferentiated hESCs, infected 1 hour earlier with either virus (or no virus, as a control), into the subcutaneous region of mice (Figure 5). Implantation of the control hPSCs, which were infected with replication-defective Ad.dE1.3 at a multiplicity of infection (MOI) of 3 or 0, resulted in the development of macroscopically large tumor nodules in 88–100% of animals within 8 weeks (Figure 5b). Histopathological analysis demonstrated that the tumor nodules consisted of various tissue types derived from three embryonic germ layers, and were therefore classified as teratomas (Figure 5c). Surv.m-CRA or Tert.m-CRA infection at the same MOI 3, which should result in adenoviral gene transduction efficiency of 66.4 ± 3.9% (Figure 5a), completely abolished tumor formation until 8 weeks after hPSC implantation. Surv.m-CRA infection a 10-fold lower MOI 0.3, which should result in adenoviral gene transduction efficiency of 27.4 ± 3.9% (Figure 5a), partially inhibited tumor formation (Figure 5b). Thus, pretreatment with Surv.m-CRA or Tert.m-CRA, which specifically and efficiently killed undifferentiated hPSCs *in vitro*, abolished *in vivo* teratoma formation in a dose-dependent manner.

## Discussion

No previous report has addressed the possibility of an oncolytic virus that could be used to inhibit hPSC-derived tumors, including teratomas. Therefore, this study represents the first demonstration of a novel m-CRA strategy that specifically and efficiently eliminates undifferentiated cells, thereby inhibiting *in vivo* teratoma formation after hPSC transplantation. Furthermore, the results of this study clearly identify Surv.m-CRA as an effective agent. Although three previously reported approaches for reduction of the tumorigenic potentials of hPSCs—reduction of the reprogramming-associated oncogenic potential of hiPSCs, purification of target cells, and generation of the engineered hPSCs—are still useful, as described in detail in the Introduction, our novel m-CRA strategy may overcome the deficiencies of these approaches. Although this method needs to be optimized in future studies using individual animal disease models, this approach should dramatically facilitate safer clinical trials of hPSC-based regenerative medicine.

The m-CRA antitumorigenic agent has several potential advantages. It should be noted that the degrees of replication of m-CRAs correlate well with the transcriptional features (*i.e*., the activity and specificity of the promoters) of the target genes. For instance, *survivin* expression levels are positively correlated with poor prognosis in human cancer patients, and the activity of the *survivin* promoter and the effectiveness of Surv.m-CRA were elevated in CSCs, which are more malignant than non-CSC fractions of cancer cells.^10,13,14^ By contrast, replication of some oncolytic viruses cannot be transcriptionally controlled. For instance, herpes simplex virus cannot always achieve cancer-specific viral replication using cancer-specific promoters, including *TERT* and *survivin* promoters.^20,21^ Thus, the highly controllable viral replication and strictly target cell-specific cytotoxicity are major advantages of m-CRA relative to several other types of oncolytic virus. Moreover, m-CRA technology allows us to further increase the specificity and efficacy by adding other cell-specific promoters and introducing transgenes, respectively.^7,8^

m-CRA also has advantages regarding safety, for several reasons. First, due to the episomal nature of adenoviruses, these constructs integrate very rarely into the chromosome. This represents a safety advantage because genomic integrations by other types of viral vectors used in clinical gene therapy have resulted in mutagenesis-derived carcinogenesis in human patients.^3^ Severe adverse side effects of such mutagenesis, including carcinogenesis, have not been clinically reported in the context of infections with wild-type adenovirus or adenoviral gene therapy. Second, wild-type human adenoviruses are not very harmful in themselves; their infections usually cause only mild and temporal symptoms, such as the common cold and epidemic conjunctivitis. Third, it should be noted that the fundamental safety of CRAs (oncolytic adenoviruses) has already been verified in several clinical trials in human cancer patients, in whom *in vivo* injections of large amounts of CRAs did not cause severe adverse side effects.^22^ Therefore, as shown in this study, it is unlikely that an m-CRA with greatly attenuated replication and cytotoxicity in normal cells would cause severe side effects when used as an *ex vivo* antitumorigenic agent in hPSC-based cell transplantation therapy.

Previous studies showed that Surv.m-CRA was one of the most promising agents for oncolytic virotherapy, for two main reasons. First, the safety and anticancer effects of *TERT*-responsive CRA had been already verified in a clinical trial, and Surv.m-CRA was superior to Tert.m-CRA in terms of both cancer specificity (*i.e*., safety) and efficiency in experiments.^8,9,22^ Second, Surv.m-CRA exhibited not only therapeutic efficacy against all populations of cancer cells, but also exerted higher efficacy against CSCs, against which conventional chemoradiotherapies are ineffective.^10^ In addition to these promising data regarding its use as an anticancer agent, the results of this study clearly show that Surv.m-CRA could be used as an undifferentiated cell-specific m-CRA agent in hPSC-based regenerative medicine. We anticipated neither that the activity of the *survivin* promoter would be so high (*e.g*., higher than the *TERT* promoter and the ubiquitously strong RSV promoter), nor that Surv.m-CRA would be more effective than Tert.m-CRA against undifferentiated normal hPSCs. This was in part because biomedical studies regarding *survivin* have focused mainly on this gene’s roles in relation to cancer,^13,14,23^ rather than hPSCs, with the notable exception of one recent paper.^15^ Therefore, future systematic analyses of candidate m-CRAs, in which viral replication is regulated by promoters of pluripotency-related and/or cancer-specific genes, would not only advance the m-CRA–based antitumorigenic strategy in hPSC-based regenerative medicine, but also help to discover novel aspects of stem cell biology.

In conclusion, m-CRAs represent novel antitumorigenic agents that should facilitate clinical applications of hPSC-based regenerative medicine. Surv.m-CRA is an especially promising agent from the standpoint of efficacy.

## Materials and Methods

### Cell cultures

KhES-1 hESCs and two lines of hiPSCs (201B7 and 253G1, here designated hiPSCs-1 and hiPSCs-2), which were generated by transduction of four (Oct3/4, Sox2, Klf4, and c-Myc) or three (Oct3/4, Sox2, and Klf4) reprogramming genes, respectively, were provided by Kyoto University through the RIKEN BioResource Center (Japan).^24^ The protocols for hESC experiments were approved by the institutional review board, followed by notification of the Ministry of Education, Culture, Sports, Science and Technology, in accordance with the Guidelines on the Utilization of Human Embryonic Stem Cells in Japan. Both hESCs and hiPSCs were grown in an undifferentiated state on mitomycin C–treated mouse embryonic fibroblasts in ES/iPS media consisting of 1:1 mixture of high-glucose Dulbecco’s modified Eagle’s medium and Ham’s nutrient mixture F-12 (Sigma-Aldrich Japan, Japan), 0.1 mmol/l 2-mercaptoethanol (Sigma-Aldrich Japan), MEM nonessential amino acids (Sigma-Aldrich Japan), 5 ng/ml recombinant human basic fibroblast growth factor (ReproCELL, Japan), and 20% KnockOut Serum Replacement (Life Technologies Japan, Japan), as described previously.^24^ The human cancer cell lines PC3 (prostate cancer), HOS-MNNG (osteosarcoma), and MKN-28 (gastric cancer), and the primary cultured HDFs were cultured in Dulbecco’s modified Eagle’s medium supplemented with penicillin/streptomycin and 10% fetal bovine serum, as described previously.^8^

To initiate differentiation, embryoid bodies were generated by the following procedure. Cells were dissociated into single cells using Accutase (Innovative Cell Technologies, San Diego, CA) in the presence of Rho-associated kinase inhibitor Y-27632 (10 µmol/l; Wako, Japan), followed by seeding of single cells at 3,000 cells/well in PrimeSurface 96-well plates (MS-9096M; Sumitomo Bakelite, Japan). Subsequently, cells were cultured for 7 days in ES/iPS media lacking basic fibroblast growth factor. Embryoid bodies were subsequently plated onto 0.1% gelatin-coated 100-mm dishes in Dulbecco’s modified Eagle’s medium supplemented with 10% fetal bovine serum (Life Technologies), and passaged using Accutase for at least 30 days before the start of experiments.^25^

### Generation of *mKate2*-expressing hPSCs with lentiviral vector (LV)

The lentiviral packaging pLenti6 plasmid (Life Technologies) and the pmKate2-N plasmid (Evrogen, Russia) were used to construct the pLenti-CA-mKate2 LV plasmid, which encodes a far-red fluorescence protein reporter gene, *mKate2*, downstream of the cytomegalovirus enhancer and β-actin promoter (CA promoter). To generate LV, 293FT cells were transfected with pLenti-CA-mKate2 plasmid and lentiviral genome plasmids (Virapower packaging mix; Life Technologies) using the X-tremeGENE 9 DNA Transfection Reagent (Roche Applied Science, Germany). LV was concentrated using Lenti-X Concentrator (Takara Bio, Japan). hESCs and hiPSCs were dissociated into single cells, and then plated onto Matrigel-coated 24-well plates (Corning Japan, Japan), followed by culture in modified Tenneille Serum Replacer 1 (mTeSR1) media (Stem Cells Technologies, Canada) for 1 day before infection. The cells were infected with LV after replacement of the supernatant with new mTeSR1 media containing 4 µg/ml Polybrene (Nacalai Tesque, Japan), and then cultured for an additional 24 hours. mKate-2–expressing hPSCs in the undifferentiated state, visualized by fluorescence microscopy, were isolated for use in subsequent experiments.

### Generation of adenovirus

The following E1-deleted replication-defective adenoviruses were propagated and purified as described previously: three types of Ads-LacZ that express *LacZ* under the control of the RSV promoter (Ad.RSV-LacZ), the cytomegalovirus immediate-early gene enhancer/promoter (CMV promoter) (Ad.CMV-LacZ), or the *survivin* promoter (Ad.Surv-LacZ); Ad.CA-EGFP and Ad.CMV-EGFP, which express EGFP under the control of the CA promoter (Ad.CAEGFP) or CMV promoter (Ad.CMV-EGFP), respectively; and Ad.dE1.3, which contains no transgene. Surv.m-CRA and Tert.m-CRA with wild-type E1A downstream of the *survivin* and the *TERT* promoter, respectively, E1B55KD downstream of the CMV promoter, and EGFP gene downstream of the CMV promoter were generated, propagated, and purified as described previously.^7,17,18^

### Promoter activities

Promoter activities were examined as described previously with some modification.^16,18^ Briefly, cells (1.8 × 10^6^ cells per plate) were incubated with Ad.Surv-LacZ, Ad.Tert-LacZ, Ad.RSV-LacZ, or Ad.CMV-LacZ at an MOI of 30 for 1 hour, and then incubated with fresh media. The cells were collected 48 hours postinfection, and β-gal activity was measured using the β-Galactosidase Enzyme Assay System (Promega, Madison, WI) as described previously.^16,18^

### qRT-PCR analysis

Total RNA was isolated using Sepasol-RNA I Super G (Nacalai Tesque) and subsequently reverse-transcribed using the PrimeScript II First Strand cDNA Synthesis Kit (Takara Bio). qRT-PCR using QuantiFast SYBR Green PCR (Qiagen, Japan) was performed on a Rotor-Gene RG-3000 (Qiagen). Relative mRNA expression levels were determined by the comparative *C*_t_ method; expression levels of individual genes were normalized using the levels of a reference gene, *hypoxanthine guanine phosphoribosyl transferase (HPRT)*. The following primer sets were used for qRT-PCR analysis at an annealing temperature of 60 °C: *survivin*, 5′-CCAGTGTTTCTTCTGCTTCAA-3′ and 5′-GAATGCTTTTTATGTTCCTCTATG-3’; *TERT*, 5′-GCCTTCAAGAGCCACGTC-3′ and 5′-AGGTGAGCCACGAACTGTC-3′; *HPRT*, 5′-TGACCTTGATTTATTTTGCATACC-3′ and 5′-CTCGAGCAAGACGTTCAGTC-3′; *mKate2*, 5′-CGTGAACAACCACCACTTCA-3′ and 5′-AAGGTTTTGCTGCCGTACAT-3′; *Lin28*, 5′-GATGTCTTTGTGCACCAGAGTAAG-3′, and 5′-CTCCTTTTGATCTGCGCTTC-3′.

### Cytotoxic effects *in vitro*

hESCs and hiPSCs were dissociated into single cells, and then plated onto Matrigel-coated 96-well plates, followed by culture in mTeSR1 media for 1 day before infection. The cells were counted and infected with Surv.m-CRA, Tert.m-CRA, or Ad.CA-EGFP at an MOI of 3 or 10 on day 0. Cell viability was determined by a WST-8 assay using the Cell Count Reagent SF (Nacalai Tesque) in accordance with the manufacturer’s protocol.^18,26,27^

### Quantitative analysis of the number of remnant hPSCs

HDFs were seeded at 10,000 cells/well in 96-well plates. One day later, *mKate2*-expressing hESCs or hiPSCs were seeded on HDFs at 1,000 cells/well. Cells in 96-well plates were infected with each adenovirus at an MOI of 3 on day 0, and image acquisition was performed on days 3, 5, and 7 using a Cellomics CellInsight high-content screening platform (Thermo Fisher Scientific, Japan), immediately after the nucleus was stained with Hoechst 33342 (Invitrogen, Carlsbad, CA). The software integrated into the screening platform accurately counted numbers of *mKate2*-expressing hPSCs (identified by magenta cytoplasm) and all cells (identified by blue nuclei), from which the percentage of *mKate2*-expressing hPSCs was calculated.

### Antitumorigenic effects *in vivo* in animal experiments

To assess the antitumorigenic effects of m-CRA *in vivo*, hPSCs were infected with Surv.m-CRA at an MOI of 0.3 or 3, or Tert.m-CRA or Ad.dE1.3 at an MOI of 3, for 1 hour, and then 3.6 × 10^7^ infected cells in phosphate-buffered saline containing 30% Matrigel were subcutaneously injected into the dorsal flanks of severe combined immunodeficient mice (CLEA Japan, Japan) (*n* = 8 for each group). The number of mice with macroscopic tumor nodules was recorded 4, 6, and 8 weeks after hPSCs implantation. Mice were sacrificed 8 weeks after hPSC implantation, and tumor nodules were collected for histopathological analysis. Resected tumors were fixed in 10% buffered formalin, embedded in paraffin, cut into 4-μm sections, and stained with hematoxylin and eosin. All animal studies were performed in accordance with National Institutes of Health guidelines and with the approval of the Division of Laboratory Animal Science, Natural Science Center for Research and Education, Kagoshima University. All reasonable efforts were made to minimize suffering.

### Statistical analysis

Data were represented as means ± standard errors. Statistical significance was determined using Student’s *t*-test. *P* < 0.05 was defined as statistically significant.
